# Influence of Water Steam on Manual Metal Arc (MMA) Welding Failure Mechanisms of Galvanized Carbon Steel Using Microstructural, Chemical, Mechanical, and Vibration Property Analysis

**DOI:** 10.1155/tswj/6146715

**Published:** 2025-10-14

**Authors:** Addisu Negash Ali, Alemkere Bayih Yizengaw, Velmurugan Paramasivam

**Affiliations:** Faculty of Mechanical and Industrial Engineering, Bahir Dar Institute of Technology, Bahir Dar University, Bahir Dar, Ethiopia

**Keywords:** experimental and computational analysis, failure modes, galvanized carbon steel, manual metal arc welding (MMAW), mechanical properties

## Abstract

The primary goal of this research is to use experimental and numerical methods to investigate the effects of water steam on the welded galvanized carbon steel mechanical properties, microstructures, and failure modes. Visual inspection, microstructural analysis, tensile test, free vibration analysis, and chemical composition analysis approaches have been used to investigate the effects of steam water treatment on the welding properties of butt arc-welded joints. From visual observation, the steam heating has significant effects in varying the microstructural arrangements of the welding. Furthermore, the elemental analysis indicates the variations of constituent elements due to the variations of steam treatment days, which affect the mechanical and microstructural properties of the material. The steam heating of the weld to a certain limit indicates the enhancement of elongation up to 20% and the amplitude during the free vibration tests. When the mechanical properties are assessed using a tensile test, the results showed that the welded specimens had up to 425 MPa ultimate tensile strength and the unwelded specimens showed up to 369 MPa ultimate tensile strength. Generally, the steam heating treatment and treatment time have significant effects on the properties of galvanized carbon steel welds. If additional treatment and controlling mechanisms can be implemented, the steam treatment will have a positive impact on the enhancement of the mechanical, corrosion, and other properties of the materials for different engineering applications. The finding of the study is that steam treatment enhanced weld ductility and tensile properties, indicating potential for augmenting weld performance in thermal cycling conditions.

## 1. Introduction

Mechanical design optimization plays an important role in engineering and manufacturing enterprises by considering its constraints which can implement practical manners in industry and factory. Most factories in the world continent use different materials with welding joints for their specific applications. In Africa, Ethiopia has various large factory sectors such as sugar factories, cement factories, soft drink factories, and textile factories, as well as facilities producing pipes for fluid/steam transportation for household applications. There are various types of joints which are permanent and temporary fasteners. Welded joint is one of the permanent joints used for making light and strong connections. They are also used to repair every weldable material and mechanical device part for recycling purposes. In designing a structure or device, more than 50,000 materials are available to the engineer.

There are several kinds of joints that serve as both temporary and permanent fastenings for different engineering applications. One type of permanent joint utilized to create strong and light connections is the welding joint. But arc-welding joints are the most common welding joints, which have versatile applications in a number of manufacturing sectors that produce sugar, cement, soft drinks, textiles, and other items used in our daily life for the movement of fluids and steam. Additionally, welding is used to repair mechanical parts and structural components using appropriate welding materials. Welding is one of the major engineering focus areas that can create job opportunities for engineers. In the welding profession, the major failure causes are lack of fusion, undercuts, and metal spattering that can lead to the formation of flaws [1].

The field visit for visual inspection and evaluation of the water steam pipes owned by Bahir Dar Textile Share Company revealed that the installed pipe was leaking steam or fluid due to the welding burst and damage. The forced water steam flow has also developed additional vibration that can accelerate the welding failure. In addition to that, the reliability of welding is reduced due to the steam heat thermal cycling of the welding joint [2, 3]. Compositions of the galvanized steel have significant impacts on the resistance of welding failure in the high-pressure steam pipes and tubing. Metals and alloys are the most widely used class of engineering materials used for steam pipes and tubes with unique structures and characteristics [4]. Ferrite-austenitic duplex stainless steels are among the many kinds of steam pipe and tube materials used worldwide. However, low alloy steel [5], Cr-Mo alloy steel [6, 7], 9Cr steels [8–10], and duplex stainless steel welding exposed to steam heat during processing and service may experience failure risks that affect the mechanical integrity and corrosion resistance. Galvanized carbon steel can be welded safely and successfully by employing crucial procedures and activities to develop high-strength steam pipes and tubes [11].

In this research, the carbon steel pipe with 1 mm thickness and 120 mm length was used in accordance with ASTM standards. As the thickness is minimum (1 mm), the welding process is very challenging, and the manual metal arc (MMA) welding technique was used to do welding on both sides [12]. The materials used for this experiment are galvanized carbon steel plates with dimensions of 120 × 13 × 1 mm. The characteristics and chemical components of galvanized carbon steel and the commonly used steam pipe material are checked using spectrometry analysis, and they are similar. By considering the results of different researchers' chemical compositions analysis, the sample material and the pipe material for industrial use have equivalent properties [13–18].

In this research, different laboratory experiments have been conducted, including analytical and compositional analysis. The use of portable spectrometers and nondestructive optical techniques is used to easily examine both the chemical and microstructural properties. Studies of the microstructural and chemical properties of arc-welded joints are applied in accordance with specific evaluation criteria [19, 20]. The most used method to characterize and determine the compositions of different metallic materials is portable ray fluorescence spectrometry [21, 22]. Because of its superior mechanical qualities and resistance to abrasion, low alloy martensitic steel is frequently utilized in wear-resistant and structural applications. Increasing the amount of antecedent deformation in samples increases their microhardness [23].

The experimental failure analysis techniques used include tensile, vibration, and composition tests [24]. Thus, the uniaxial cross-weld test of welded joints with respect to parent material, weld material, and heat-affected zone (HAZ) materials can help to characterize the mechanical properties and provide real insight into the evolution of stress–strain and other thermomechanical properties [25–30]. Cracks, porosity, undercut, slag inclusions, overlap, incomplete fusion, incomplete penetration, intraluminal failure, interfacial failure, and coupon failure are among the many failures/defects that can occur on welded joints both during service conditions and after a lengthy service life [31, 32]. Therefore, failure mode analysis is the best approach to develop solutions for steam-heated galvanized steel materials by identifying/detailing the sorts of failures or flaws and characterizing their mechanical properties [33–35]. Furthermore, for a proper production process or service condition, time and fluid loss must be minimized during welding [36–39].

Different literatures have discussed mechanical properties and cracking or fracture conditions, show analysis or study of specific arc-welding type's failure assessment and determination of its mechanical properties at different materials, and address the welding joint microstructural composition of materials. Through the above review of related research, works show that the welding joint has much potential as a main joint in various sectors such as structural, automotive, aerospace, and marine applications. Welding is the main joining fastener used in different industries/factories for the joint of materials and can lead to failure or burst components during service life/after some time. It needs to maintain the welding joint to increase the duration of the maintenance period through failure analysis techniques and obtain better information about materials. However, owing to their recent discovery, not much research has been done on the welding joint with environmental relations. In this study, the effects of variable weather/fluctuating weather environments on galvanized carbon steel with produced steam and predictable failure modes are shown. The main objective of this research is the influence of water steam on MMA welding failure mechanisms of galvanized carbon steel using microstructural, chemical, mechanical, and vibration property analysis.

## 2. Materials and Methods

### 2.1. Materials

For these experimental studies, the galvanized carbon steel plates having the 120 × 13 × 1 mm sizes are used as the base metal. Tensile test samples have a chemical element with different percentages, that is, the chemical composition of Mn (0.12%), Fe (99.88%), Ti (0.23%), V (0.1%), Cr (0.12%), Co (0.27%), and others.

### 2.2. Welding Methods

The duration of most MMA welding actually makes it necessary to employ different weld beads for each pass. Before welding, the base metal is thoroughly cleaned with acetone using a line-free cloth. To prevent misalignment between the plates, the base metals were welded on both ends and properly clamped before the welding was finished. Although there are several butt arc-welding preparations, including single v-butt, single u-butt, double v-butt, and double u-butt joints, a very thin plate material was used for this investigation with one side butt instead of v-butt or u-butt. Butt joints are defined for this study as those that exist between plate edges, which are often in the same plane. A flux is applied to the electrode melts and covers the molten metal to provide protection. Based on the specifications information in the market, the MMA welding electrodes had lengths between 200 and 450 mm and diameters between 1.5 and 8.0 mm; for this study, a 2.5 mm diameter and 300 mm length electrode was used. The AWS E6013 electrode, which has a diameter of 2.5 mm, is utilized for the welding process in an MMA welding machine. The area of the weld-melted zone on a plane perpendicular to the welding direction of motion can be used to estimate the welding heat input. Furthermore, the weld's strength is determined by the process conditions.

As indicated in Tables 1 and 2, the following parameters were used for this study project: dia = 2.5, length = 300, and flat = 60–90. Although the actual current taken varies slightly between 22, 25, 27, and 31 amperes, the average current used is 26 amperes determined by considering the thickness of the specimens. The welding size is 500 mm in length, and the welding time taken is *t* = 4 min. Vt = *S*/*t* = 500 mm/4 min = 125 mm/min is its traversal speed, which is 3.25 mm/min or 0.054 mm/s for a single specimen.

For this investigation, AWS E6013 and galvanized carbon steel were utilized as consumable electrodes with a diameter of 2.5 mm and base metal, respectively. The test samples' test welded joints and beads were completed on 250 × 6 × 1 mm lengths. Despite being more common than metal inert gas welding, MMA welding is still frequently utilized since it was the first of many welding methods to use an electric arc as a heat source. Executive standard GB15579.1-2004 method was used to manufacture the MMA welding machine under study. The model MMA-200 with an inverter MMA 30A/21.1–200A/28 V power supply, a power factor of −0.86, a frequency of 50/60 Hz, and a fan cooling system was used. The recommended current and the chosen value are similar with the welding machine specifications. The mechanical properties of the base galvanized metal and weld galvanized metal are listed in Table 3.

### 2.3. Specimen Shape and Geometry

The material under consideration is galvanized carbon steel plate, which is 1000 × 2000 mm and has a thickness of 1 mm. To compare the newly prepared weld material model, both side pass welding models were used. In order to prepare the necessary specimen from galvanized sheet metals, different machining processes were conducted using different mechanical tools. These instruments include cutting machines, shearing machines, drilling machines, scrapers, steel rules, calipers, and C-clamps. With the aid of a shear cutting machine, samples for mechanical testing were machined from the weld specimen in a direction transverse to the weld bead. Following the shear cut of the entire plate, weld beads for the sample were created on 250 × 6 mm samples. Galvanized carbon steel longitudinal tensile specimens were cut both before and after welding in order to test for tensile, vibration, microstructure, composition, and digital image correlation (DIC).

**Figure 1:**
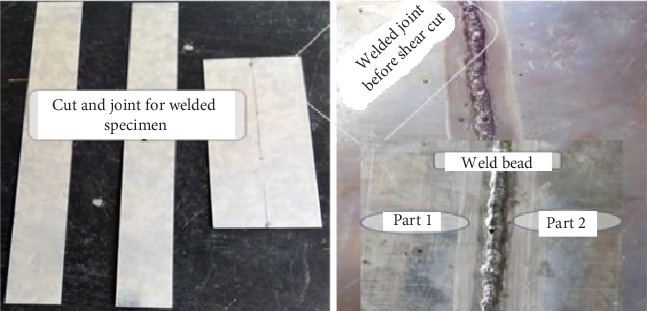
Welding specimen preparation before shear cut.

**Figure 2:**
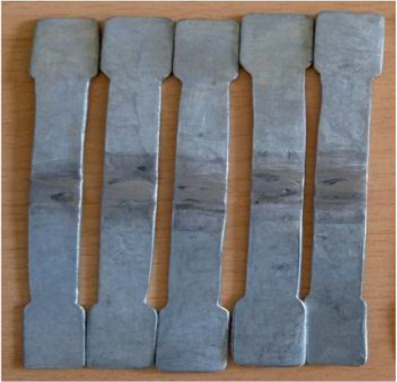
Final welded specimen preparation for tensile test.

Using specimens that were 120 mm long and 10 mm wide, the size of the specimens was determined proportionately, and the soundness of the weld joints was examined in accordance with ISO 6892-1 and ASTM E8-2016 standards. Tensile test specimens are constructed in accordance with ASTM standards (ASTM A506) and evaluated using UTM. The ultimate tensile strength and yield stress of the welded joints are ascertained through tensile testing. As shown in Figures 1 and 2, sample preparation involves making minor adjustments to a flat piece of circular pipe. Galvanized carbon steel was used to create flat tensile specimens that were 80 mm gauge length, 10 mm wide, and 1 mm thick. The tensile test was conducted using a universal testing machine button adjustment with a cross head displacement rate of 1 mm/min.

### 2.4. Metallography Analysis Specimen Preparation

The metallographic analysis was conducted on specimens before and after the welding process. SiC paper with grit sizes of 400, 600, 800, and 1000 was used to polish the samples, which were etched with 99.5% concentrated acetic acid. After dipping the sample in acetic acid for approximately 15 s, all polished specimens were etched and allowed to dry in the open air. For metallographic analysis, the sample was polished and etched in a plane perpendicular to the longitudinal and circumferential welds. To achieve a scratch-free surface, each of these samples was mounted separately in conductive mounting and polished using standard metallographic processes. A thermomechanical impacted zone was discovered as the longitudinal microsection was being etched. A tiny sample measuring 6 × 7 × 1 mm should be cut for cross-sectional morphological assessment. Before welding, the work parts' surfaces are thoroughly cleaned to get rid of oil and debris. In this experiment, 20 V of welding voltage, 150 A of welding current, and 0.054 mm/s of welding speed were employed. SiC abrasive papers were used to clean and massage the surface of the carbon steel material. Distilled water was then used to wash it, and then ethanol alcohol. The voltage and frequency of the cooker and stoves are the same (202–240 V, 50/60 Hz); however, the voltage was varied. Their maximum temperature and power during steam heating were 240°C and 1000 W, respectively.

### 2.5. Microstructure Analysis

One of the most effective methods for identifying functional groups of chemical compounds is Fourier transform infrared (FTIR) spectroscopy. Its function is explained or highlighted by the use of FTIR spectroscopy to identify the chemical components by determining the variety of functional groups. By generating an infrared absorption spectrum, FTIR spectroscopy can determine the chemical bonds present. FTIR is a technique that measures absorption from an infrared light source to identify compounds. As a consequence, the FTIR-6600 Type A model, Serial Number Ao13861790, light source standard, 3736 data points, scanning speed of 2 mm/s, and filter frequency of 10,000 Hz are utilized for this investigation. With a spectral resolution of 4 cm^−1^, a scanning speed of 2 mm/s, and a maximum sampling frequency of 10,000 Hz, it covers the 4000–400 cm^−1^ or 1.8–8 *μ*m range.

### 2.6. Chemical Composition Analysis

A portable spectrometer is used to examine the chemical compositions of the welded specimen, and galvanized carbon steel is as shown in Table 4. A welded sample was etched to ascertain the microstructure and machining location of specimens in order to analyze the local composition using a portable spectrometer. Using a portable spectrometer in accordance with ASTM E975, the elemental composition of the sample was evaluated using eight specimens and one pipe material as a benchmark parent material with a dimension of 120 × 10 × 1 mm. For determining the abundances of major and minor elements, the portable spectrometer's typical relative uncertainty is three. Its precision ranges from Mg to U for all elements. The portable spectrometer's small sample area contact focus region was used to make the measurements. With 50 kV excitation sources, the portable spectrometer equipment can detect solid and powder particles up to 25 mm and measures elements in the Mg–U range using the x-ray fluorescence analysis method.

### 2.7. Experimental Tensile Test

**Figure 3:**
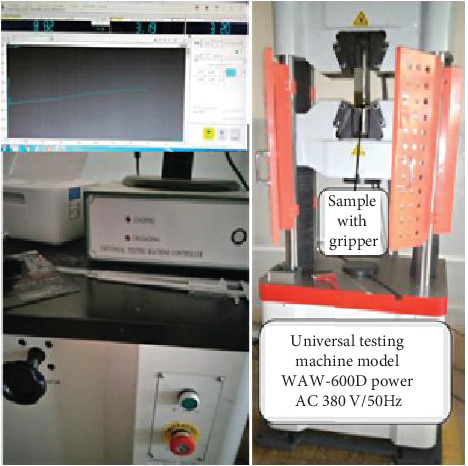
Tensile strength test experimental setup.

Tensile test specimens were made in accordance with ASTM E8M-16a; each sample had a thickness of 1 mm and a width of 10 mm at the reduced cross-section. A universal testing machine (WAW-600D) was used to perform tensile tests at room temperature with a strain rate of 1.0–2.0 mm/min. A tensile test machine with a 10 kN load capability was employed. The initial gauge length was set at 68–72 mm, and the cross head displacement was set at 1 mm/min.  Figure 3 illustrates that the machine's average applied load is 6.51 kN. To examine the fracture surfaces using an optical microscope, clear acrylic was applied and then removed with acetone. Tensile testing was done between 4.84 and 8.82 kN. Tensile tests were performed on cross-welded samples of base steel at room temperature of approximately 20°C to determine yield strength, tensile strength, and elongation at the welded joints. Every sample was made in accordance with ASTM standards and dimensionally reduced by a percentage to match the dimensions of the entire experimental setup. In general, the following formulas were utilized to analyze the experimental data.  Figure 3 illustrates the tensile strength experimental setup to determine the highest strength of the material.

### 2.8. Optical Microscope Observation

**Figure 4:**
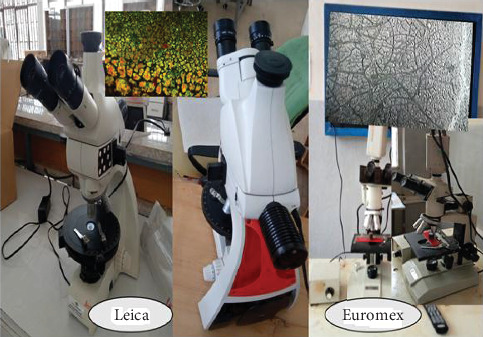
Optical microscope microstructure measurement setups.

Following the metallographic etching and mechanical polishing, optical microscopy analysis was used to characterize the microstructure of welding joints encountered as shown in Figure 4. Shear cutting was used to prepare the sample of the metallographic specimens in the welded zone, and metallographic sandpaper was used for grinding and polishing. Sandpaper grinding, water washing, ethanol acid degreasing, and alcohol clearing were all completed, dried, and set aside prior to welding. Leica and Euromex optical microscopes were used to examine the microstructure of the weld joint and nonwelded specimens. When viewed under a nontransparent optical microscope, the sample's microstructure reveals surface patterns and metallurgical analysis. A nontransparent microscope was used to assess the difference in microstructure between the welded and nonwelded zones in order to characterize the microstructure.

### 2.9. The Vibration Behavior Measurement Setups

**Figure 5:**
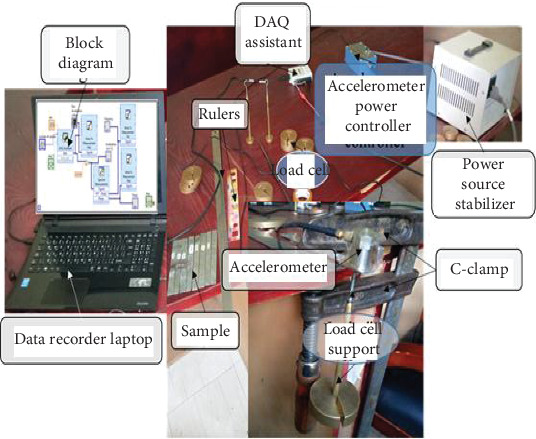
The vibration analysis experimental setups.

A manual exciter is used in a laboratory vibration test to determine the frequency of the prepared samples as shown in Figure 5. To investigate the samples' inherent frequencies, the modal analysis was carried out experimentally. In a mechanical system, oscillation is determined by the amplitude and frequency (frequencies). Calculating or measuring the inherent frequencies of mechanical systems is sometimes the most crucial component of vibration analysis. The excitation force can be impact, periodic, sinusoidal, or random.

When feasible, a force with enough energy and frequency components should be used in practice to excite all vibration modes of interest and to minimize signal processing challenges, which will result in the compilation of accurate FRF data. A device called Hammer generates an excitation force pulse for the study's test structure. The plastic head is used for stimulation. The most used sensor for modal testing is an accelerometer. It produces the signal as voltages and measures the acceleration of a test structure. The linearity and other characteristics of the observed structure are not assumed by the accelerometer. A spring and a damper are used to model installation, which has a significant impact on the acceleration measurement's accuracy. The signal-to-noise ratio of an accelerometer is determined by its sensitivity. Following test stimulation by a known load cell, failure analysis was conducted. It is possible to idealize a number of structural and mechanical systems as single-degree-of-freedom systems. Although the mass is distributed in many real-world systems, it can be roughly represented by a single point mass for straightforward analysis. Likewise, a single spring might idealize the system's elasticity, which may be dispersed across the system. The single-degree-of-freedom model for transverse vibration can be obtained by approximating the supporting structure (beam) as a spring and treating the top mass as a point mass. An input fast Fourier transform (FFT) curve was used to construct the random free vibration test, which was carried out at a frequency of 25,000 Hz using 2000 sample readings.

## 3. Results and Discussions

### 3.1. Visual Inspection of Sample

**Figure 6:**
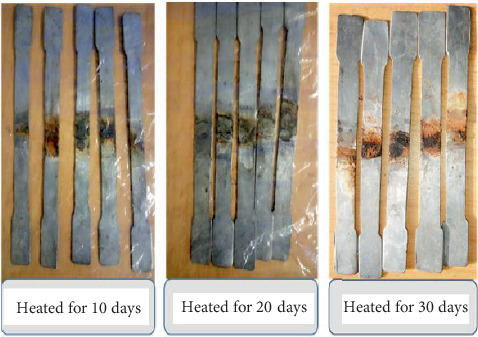
Welded specimens after steam heating for tensile test.

**Figure 7:**
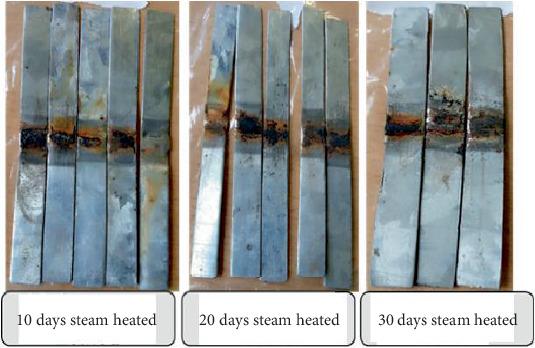
Welded specimen after steam heated for vibration and microstructure analysis test.

The detailed observation of the fracture surface indicates differences in fracture surface topography, reflectivity, and color characteristics. Photography is used to record the features and conditions seen during a failure analysis examination. At 1–8× magnification, the blue gray covering looked thick, and the rust-colored oxide was impossible to remove. The welded specimens in the macroshot exhibit corrosion following varying heating times (i.e., 10, 20, and 30 days of steam heating), as illustrated in Figures 6 and 7. Due to the formation of numerous layers after 10, 20, and 30 days, the corrosion process began with exposure at 170°C after the start time. After being exposed for 10, 20, and 30 days, the surface of the welded specimens underwent a significant alteration. The temperature at the metal-corroding contact must be taken into account when examining how temperature affects a metal's rate of corrosion in a liquid or gaseous environment. Under harsh circumstances, which are frequently present in environments, carbon steel material is damaged. This material's degradation poses a threat to biological variety, human life, and the industrial sector's financial standing. According to earlier research, the passive condition of carbon steel was investigated and deteriorated when exposed to the environment at a constant corrosion rate. This indicates that the corrosion characteristics of iron or carbon steel in concrete water solutions are influenced by temperature. The 30 tested samples are shown in Figures 6 and 7.

One significant factor that alters the corrosion properties of carbon steel surfaces is temperature, which has an impact on all electrochemical corrosion mechanism processes on carbon steel surfaces. Consequently, there are significant differences in the four welded specimen cases, as evidenced by visual inspections, numerous failure cases, and crack initiation brought on by a corroded layer or surface.

### 3.2. Analysis of the Welded Joint Microstructure

**Figure 8:**
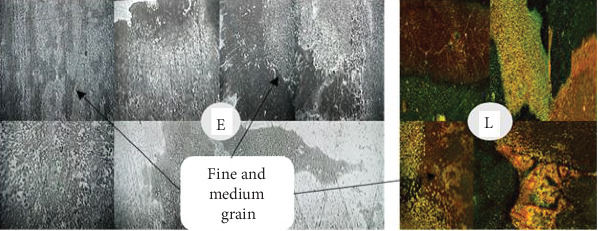
Welded unheated specimen 10×.

**Figure 9:**
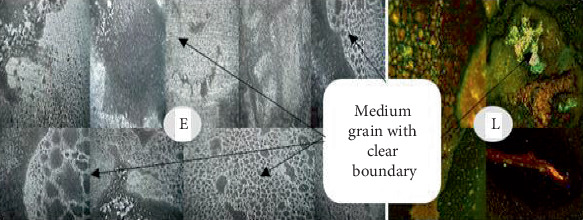
Weld with 10 days' heated specimen 10×.

**Figure 10:**
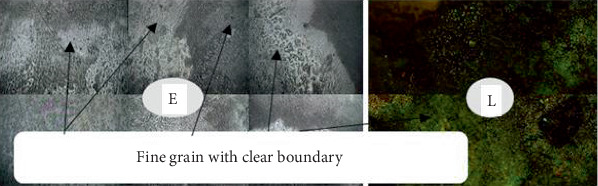
Weld with 20 days' heated specimen 10×.

**Figure 11:**
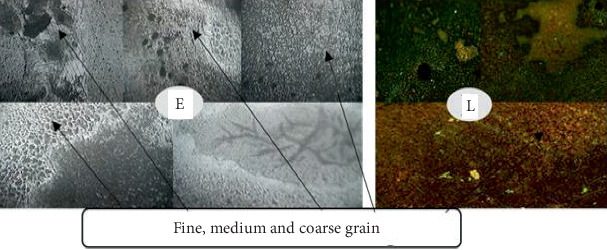
Weld with 30 days' heated specimen 10×.

Following the completion of the steam heating operation, the microstructure was examined for each sample. The microstructure analysis revealed that, following the steam heating operations, the carbon steel has the ferrite and pearlite microstructures. As seen in Figures 8, 9, 10, and 11, the carbon steel's microstructure at the same magnification (10×) showed a distinct pattern.

As shown from Figures 8, 9, 10, and 11, fine- and medium-sized microstructures are visible in unheated welded specimens; however, a medium grain boundary is clearly visible in specimens that have been heated for 10 days. Additionally, specimens heated for 20 days display fine grain with a distinct boundary, whereas specimens heated for 30 days display both fine and coarse grain with a distinct boundary. As a result, two types of grain architectures are observed in the four distinct examples. Between the unheated case and the 30-day hot welded specimens, the grain size increased. Consequently, there is a significant variation in the types of welded specimens across the four successive stages, indicating that unheated weld specimens are more resistant to failure than 30-day hot weld specimens.

### 3.3. Chemical Composition Analysis

The recorded results in this investigation are displayed in Table 5 which showed the impact of elements on welded specimens in four distinct scenarios. Following the heating of the specimens by the steam, the studied samples were cross-sectioned and polished using typical metallographic procedures. Iron, manganese, titanium, vanadium, chromium, and cobalt are the fundamental elements that are present in the reported data, as indicated in Table 5. Manganese exhibits significant variation between unheated and 10-day heated specimens. The manganese weight percentage decreases in 20-day heated specimens and increases in 20–30-day heated specimens. This element has the effect of decreasing the strength of steel specimens heated for less than 20 days while increasing the strength of welded specimens that are heated for 20–30 days. For welded specimens, there is a minimum variation of iron weight percentage between unheated and 30-day heated specimens, although the iron weight percentage increases from unheated to 20-day heated specimens. From the unheated case to the 20-day heated case, the solidification effect increases; however, at the 30-day heated case, the strength and minor solidification diminish.

Vanadium and titanium have the same impact on welded specimens, although cobalt and chromium differ slightly. Titanium and its alloys are said to have the same mechanical qualities as other metals; however, they are utilized to increase corrosion resistance in unheated situations while maintaining the same effect in other applications. The chromium weight percentage increases from unheated to heat for 10 days, but the next heating conditions, such as 20 and 30 days, have the same impact. Compared to unheated conditions, weld specimens exhibit greater strength at elevated temperatures. When compared to unheated welded specimens, small content elements diminish after 20 days of heating, but they start to appear after 30 days of heating. There is no indication of the percentage of carbon in the recorded data and explanation. Nonetheless, earlier research showed that irons, which exist in two crystal forms below the melting temperature, make up a significant portion of steel. Carbon is also present in steels in varying levels, from 0.005 wt% in ultralow carbon to 2.00 wt% in the highest carbon tool steel [18].

The following are the impacts that alloying elements have on carbon steel. Between unheated and 20-day heated specimens, Mn increases strength with decreasing brittleness; however, for 30-day heated welded specimens, Mn decreases strength with increasing brittleness. In contrast to increasing hardenability from 10 days heated to 20 days heated and decreasing to 30 days heated, Cr was utilized to decrease hardenability from unheated to 10 days heated. However, it enhances hardenability from unheated welded to 10 days and decreases hardenability from 10 days heated to 20 and 30 days heated. It also significantly reduces hardenability at unheated case from welded to welded specimens. Light elements increase hardness to 30-day heated specimens and decrease hardness from unheated, unwelded specimens to 20-day heated specimens. They also slightly raise hardness to 30-day heated specimens without surpassing unheated welded examples.

### 3.4. Steam Heat and Functional Groups

**Figure 12:**
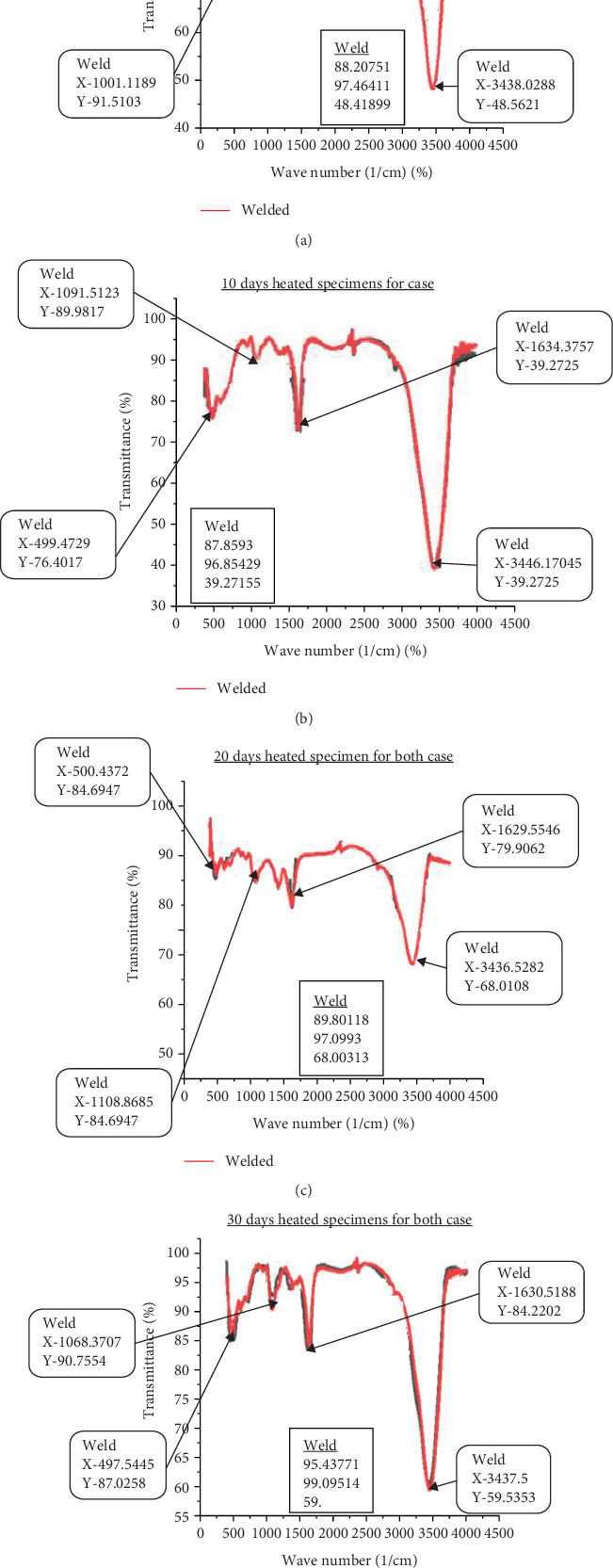
(a–d) Comparison between individuals.

The FTIR spectra have different peaks related to the existence of CH_2_, C≡N, C=O, and C-O and other compounds due to the steam segregation in the weld, which has a significant effect on the formations of corrosion and other failure causes. A peak at 2916 cm^−1^ is mainly because of C-O bonds. Another peak at 2243 cm^−1^ may be the indication of nitrile (C≡N) structure. The spectra corresponding to 1668 cm^−1^ are attributed to the cyclic C=O structure, while the peak at 1456 cm^−1^ is mainly due to the CH structure [40]. In this study, transmittance (%) versus wavelength (cm^−1^) explains the existence of different microstructures related to steam heat. As Figure 12 makes evident, the transmittance fluctuates on the graph in relation to wavenumber, supporting theories about the formations of different compound structures. There are four distinct examples of steam heating for weld specimens in this study: unheated, 10, 20, and 30-day steam heated.

**Figure 13:**
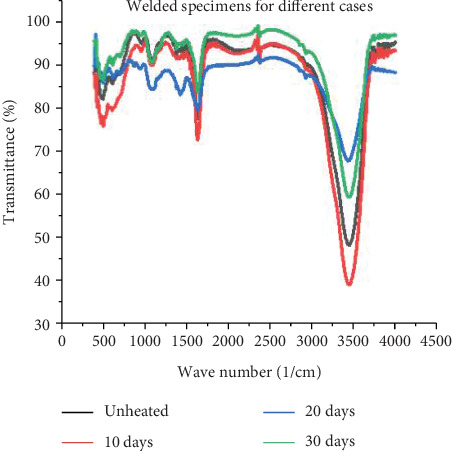
Comparison between unwelded and welded specimens.

The formations of compounds mostly relate to specimen transmittance as shown in Figure 13. Compared to the other three examples, which show 97% transmittance at start time with intervals of 97%–48%, welded unheated specimens have a weaker connection. The transmittance of welded specimens heated for 10 days begins at 81% and ranges from 97% to 40%. The transmittance of welded specimens heated for 20 days begins at 89% and ranges from 97% to 68%. Specimens that have been heated for 30 days begin with 95% transmittance and range from 98% to 59%. This suggests that the structure of welded specimens decreases from the unheated case to 10 days of heating, but increases significantly to 20 and 30 days of heating. Since transmittance and wavenumber are inversely correlated with compound structure, a higher transmittance % corresponds to a weaker compound and a lower transmittance to a stronger compound. Thus, this study demonstrates that small steam has a greater influence on strength loss.

### 3.5. DIC

**Figure 14:**
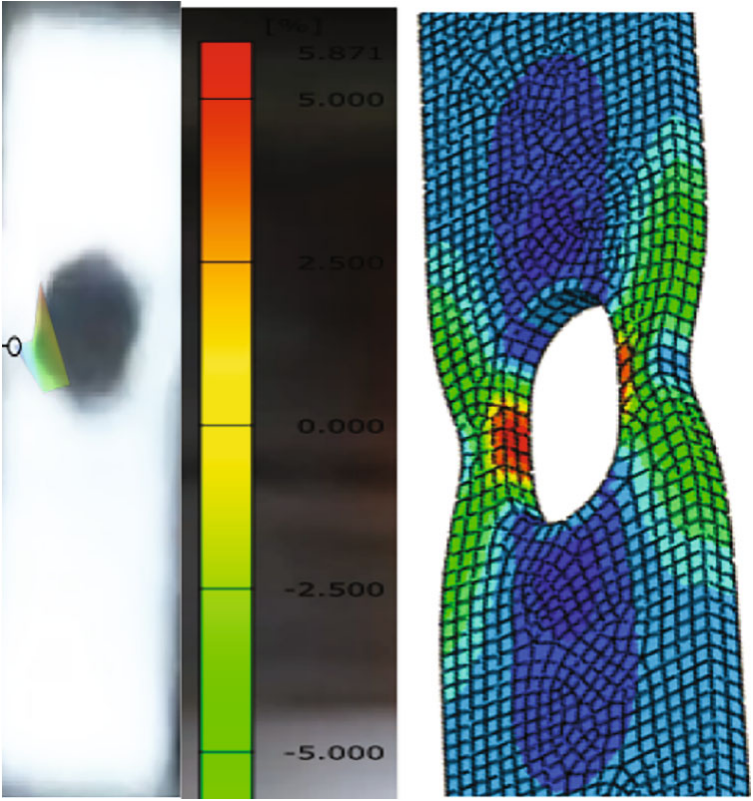
Major strain of welded unheated specimen.

**Figure 15:**
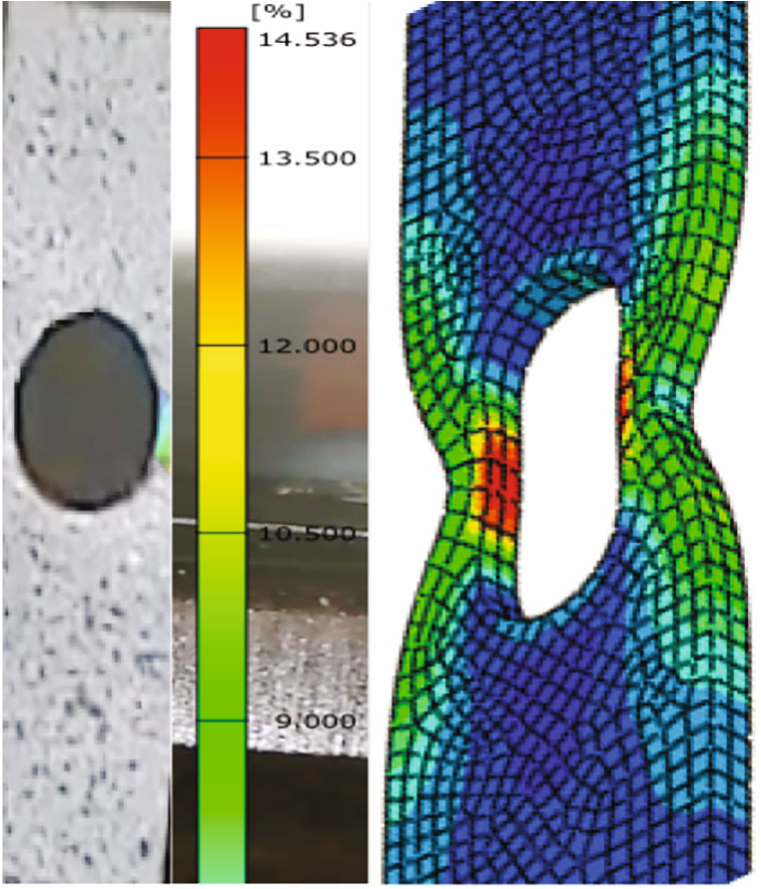
Major strain of welded 10-day heated specimen.

Through the use of the DIC technique, an experimental investigation into the load-carrying and deformation capacities of galvanized carbon steel butt-welded joints under tension load is conducted in order to examine the development of the strain distribution over the specimen during the entire loading process, as shown in Figures 14 and 15.  Table 6 reveals that the specimens had varying analytical elongation ranges. The welded joint with 20–30-day steam-heated specimen showed a significant change of elongation of 7%–20% and 13%–17%, respectively, which is higher compared to the unwelded specimens.

**Figure 16:**
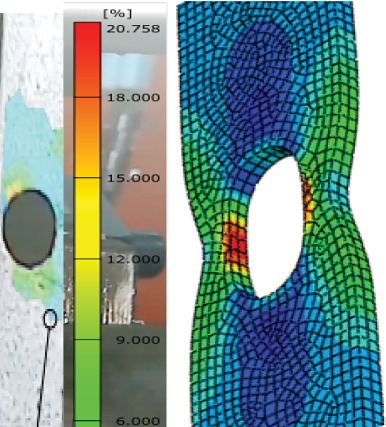
Major strain of welded 20-day heated specimen.

**Figure 17:**
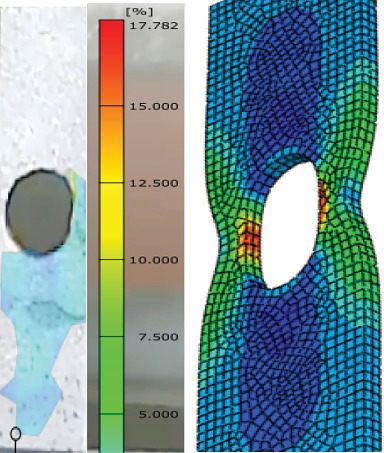
Major strain of welded 30-day heated specimen.

The GOM software study yielded a number of significant strain values. The welded specimens with unheated conditions showed the major strains of 5.871%, while 14.76% elongation was achieved after 10 days of heating, 20.76% after 20 days of heating, and 17.78% after 30 days of heating. For welded specimens, the strain gradually decreases after 30 days of heating. Actually, all four cases at the hole corner showed significant strain for both types. This situation was explained by the fact that concentration of strain causes failure around welded joints that have HAZs and weld beads, as shown in Figures 16 and 17. The longer the steam heating, the more deformation occurs, and the material's elongation increases. Compared to unheated weld specimens, weld specimens fail as distortion increases.

### 3.6. Tensile Test Result

**Figure 18:**
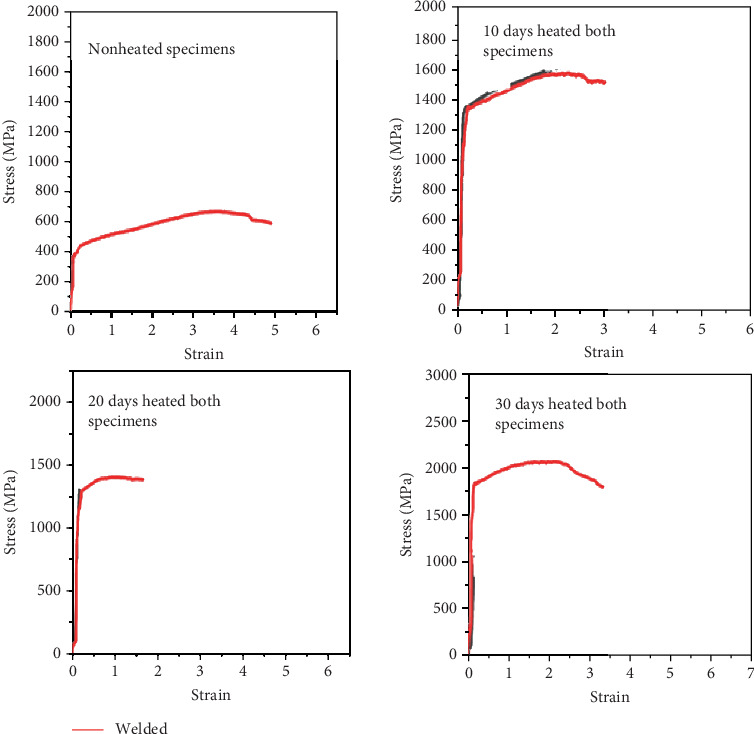
Comparison between unwelded and welded specimens with strain–stress diagram.

**Figure 19:**
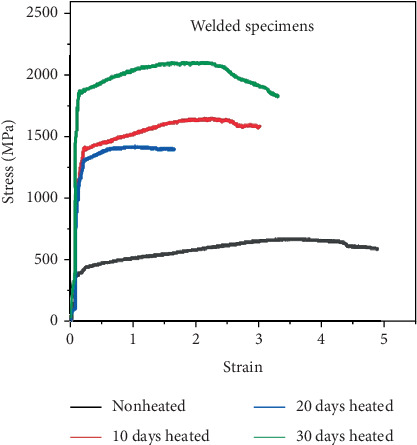
Tensile test comparison between welded and steam-heated specimens.

As observed from the results obtained, the mechanical properties of the welded specimen are significantly dependent on the steam heating. Figures 18 and 19 show the experimentally recorded values for the tensile strength of galvanized carbon steel welded joints in four distinct scenarios. Compared to the unheated welded specimens, heated welded specimens have a very high strength. In comparison to specimens cooked for 10 days, those heated for 30 days have a higher yield stress. The stress–strain relationship after 10, 20, and 30 days demonstrates that weld specimens fail before and below unheated weld specimens. All tensile characteristics showed that the transverse-to-the-weld specimen and the longitudinal weld varied from 291 to 491 MPa and 121 to 208 MPa, respectively, and elongations from 7% to 20%. Thus, the aforementioned findings demonstrate the inferred comparability of weld specimens using tensile testing under various scenarios.

### 3.7. Free Vibration Analysis

**Figure 20:**
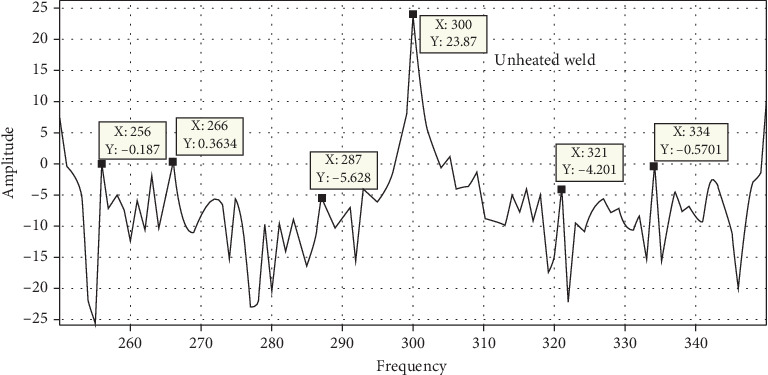
MATLAB filtered result for unheated weld specimens.

**Figure 21:**
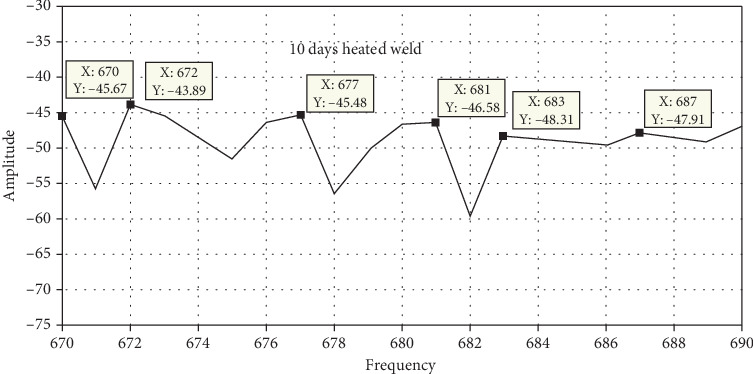
MATLAB filtered result for 10-day heated weld specimens.

**Figure 22:**
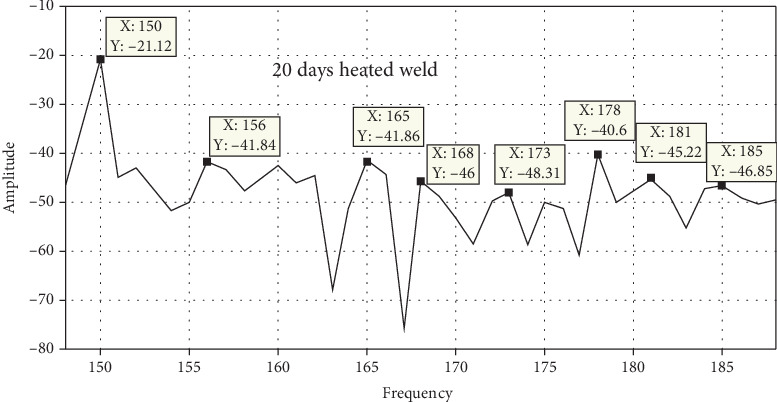
MATLAB filtered result for 20-day heated weld specimens.

**Figure 23:**
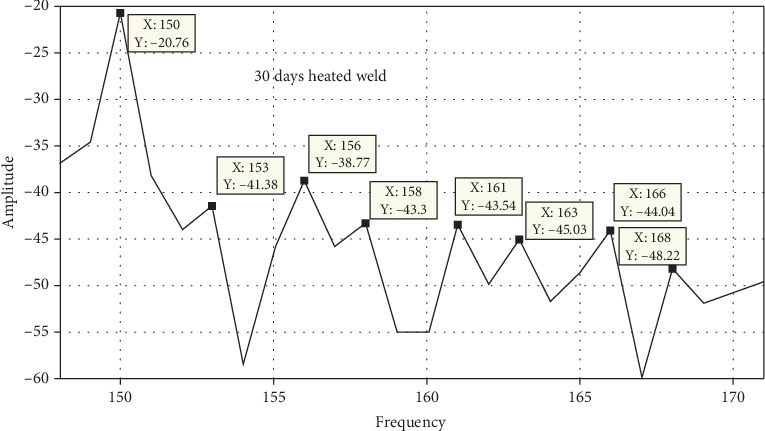
MATLAB filtered result for 30-day heated weld specimens.

**Figure 24:**
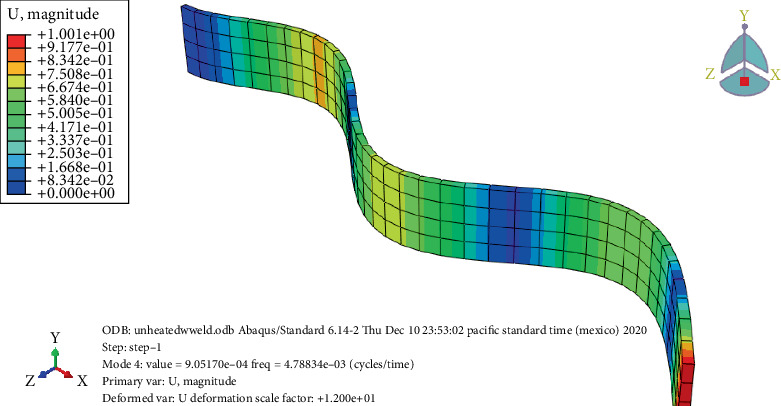
Unheated welded specimen frequency.

**Figure 25:**
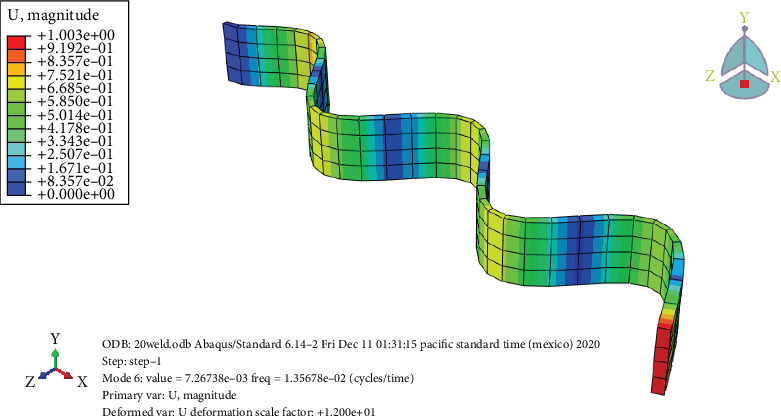
The 10-day heated welded specimens' frequency.

**Figure 26:**
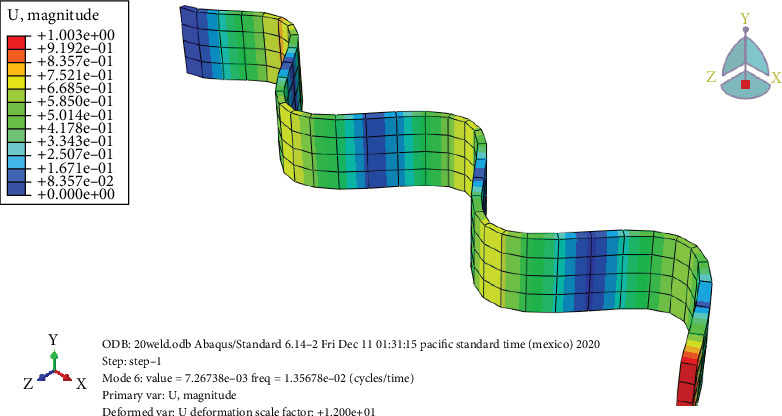
The 20-day heated weld specimens' frequency.

**Figure 27:**
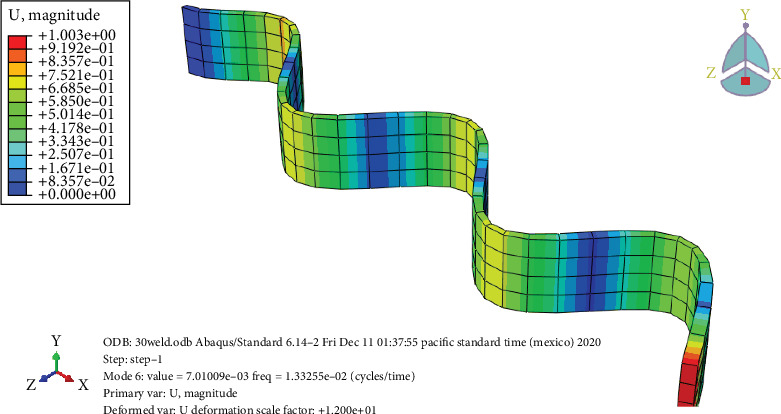
The 30-day heated welded specimens' frequency.

Static brittle fracture is the primary failure mechanism, according to a comparison of the surface broken during vibration testing. Frequency response is used to determine the stress states and failure critical location. Amplitude versus time, frequency versus time, and phase angle versus time are used to examine the failure mechanisms and locations as shown in Figures 20, 21, 22, and 23. A 120° × 13 carbon steel beam is used for testing. It is fixed as a cantilever, and excitation is applied using a hammer excited. The beam's behavior is recorded as modal analysis to determine the natural frequency and various mode shapes as observed in the experimental setup. Some of these have been confirmed using Abaqus software as shown from Figures 24, 25, 26, and 27, which allows the theoretical value to be compared to the experimental value [41, 42].

Unheated and 10-day heated welded specimens of frequency response with respect to amplitude value have the same effect, but the 10-, 20-, and 30-day heated specimens have a higher amplitude value. These indicate that weld specimens are less deformed compared to the 10-, 20-, and 30-day heated cases.

Figures 24, 25, 26, and 27 and Table 7 show that the unheated case's mode changes to a 10-day heated weld specimen and the weld specimens' mode changes from 4 to 6, while the temperature remains constant from 10 days heated to 30 days heated.

## 4. Conclusions

• Metallurgical investigations have been performed on all four cases of samples in order to determine failure mechanism, microstructure, and chemical properties. Chemical and metallurgical changes involved in the welding process are critical considerations in the development of welding procedures as well as joint design methods.
• Alloying elements have distinct effects on carbon steel. Mn decreases strength, Cr decreases hardenability, and light elements decrease the hardness of welded specimens, while in most cases, they have a lesser effect on unwelded specimens. Most cases of specimens indicate that welded specimens have lower bond strength compared to unheated welded specimens.
• Therefore, light elements in welded unheated specimens have great value compared to unheated welded specimens, but this value decreases in 10-, 20-, and 30-day heated specimens. This indicates a reduction in the hardness of heated specimens compared to unheated specimens.
• The iron percentage increases hardness for unheated welded specimens, but reduces in other welded specimens.
• The metal arc–welded specimens underwent a variety of tests, including visual inspection, microstructural analysis, tensile testing, and vibration analysis.
• The water steam heating to a certain limit leads to the formations of compound structures that affect the mechanical properties of the weld.
• The yield strength of the weld specimen improved due to the steam heating for a certain limit.
• The deformation (elongation) of the weld specimen increased when increasing the steam heating days of the sample.
• The weight percentages of the alloying elements varied over different steam heating days, affecting the mechanical properties and corrosion resistance of the material.
• Tensile tests showed ultimate tensile strength of welded specimens up to 425 MPa, compared to 369 MPa for unwelded ones. Yield strength reached 208 MPa for welded samples. Elongation increased with steam heating (up to 20%), indicating higher ductility after prolonged treatment.
• Visual inspection showed corrosion and crack initiation intensified with longer steam exposure. DIC revealed higher strain concentrations at weld zones, with deformation increasing after extended heating. Free vibration analysis showed mode changes, and amplitude increases for heated welds, indicating reduced stiffness and higher susceptibility to dynamic failure.
• The primary focus of this study is to obtain better information on welding joint failure. Future researchers should examine individual parts of welded samples, that is, different regions of the weldment like base metal, HAZ, and weld metal, to compare and analyze them effectively. This research might be effective in a more isolated environment using a steam-heated experimental setup. The research will yield sound microstructural results if the samples are observed through TEM and EBSD.

## Figures and Tables

**Table 1:** Experimental settings for the welding process preparation.

**S. no.**	**Welding method**	**Filler material**	**Current (A)**	**Voltage (V)**	**Welding traveling speed (mm/s)**
1	MAW	AWS E6013	26	20	0.054

**Table 2:** Recommended welding current for the used electrode.

**Size (mm)**		**Current range (A)**	
**Diameter**	**Length**	**Flat**	**Vertical and overhead**
2	300	40–60	30–55
2.5	300	60–90	50–80
3.2	350	90–130	80–120
4	400	140–190	120–170
5	400	190–240	150–200

**Table 3:** Mechanical properties of the base galvanized metal and weld galvanized steel.

**Materials**	**Tensile strength (MPa)**	**Yield strength (MPa)**	**Elongation in 50 mm**	**HRB**
Base galvanized steel	310–385	235–290	30–38	47–68
Weld galvanized steel	305–350	220–270	32–40	42–54

**Table 4:** Chemical composition of the base galvanized metal and weld galvanized metal (wt%).

**Element**	**Mn**	**Fe**	**Ti**	**V**	**Cr**	**Co**	**Ni**	**Cu**	**Zn**	**Zr**	**Nb**	**Mo**
Base galvanized metal	0.12	99.88	0.23	0.1	0.12	0.27	0.02	0.01	0.01	0.01	0.01	0.01
Weld galvanized metal	0.23	99.71	0.22	0.11	0.061	0.27	0.02	0.01	0.01	0.01	0.01	0.01

**Table 5:** Chemical compositions of welded specimens at different steam treatment conditions.

**Element**	**Mn**	**Fe**	**Ti**	**V**	**Cr**	**Co**	**Ni**	**Cu**	**Zn**	**Zr**	**Nb**	**Mo**
Unheated	0.23	99.71	0.22	0.11	0.061	0.27	0.02	0.01	0.01	0.01	0.01	0.01
10 days	0.21	99.79	0.23	0.1	0.12	0.27	0.02	0.01	0.01	0.01	0.01	0.01
20 days	0.16	99.84	0.23	0.1	0.11	0.28	0.02	0.01	0.01	0.01	0.01	0.01
30 days	0.28	99.72	0.23	0.11	0.11	0.28	0.02	0.01	0.01	0.01	0.01	0.01
**Element**	**Ag**	**Cd**	**Sn**	**Sb**	**Hf**	**Ta**	**W**	**Re**	**Ir**	**Pt**	**Au**	**Pb**
Unheated	0.09	0.1	0.17	0.28	0.07	0	0.03	0.04	0.06	0.02	0.01	0.05
10 days	0.09	0.09	0.16	0.26	0.05	0	0.03	0.04	0.06	0.02	0.01	0.05
20 days	0.08	0.09	0.16	0.25	0.05	0	0.03	0.04	0.06	0.02	0.01	0.06
30 days	0.09	0.09	0.17	0.26	0.07	0	0.03	0.04	0.06	0.01	0.02	0.05

**Table 6:** Elongation values in %.

**Type**	**Not welded**	**Welded**
Unheated	12–18	12–14
10 days heated	14–24	10–14
20 days heated	9–17	7–20
30 days heated	12–17	13–17

**Table 7:** Summary result of mode and frequency.

**Type**	**Case**	**Mode**	**Frequency (Hz)**
Weld	Unheated	4	4.788
	10 days	6	1.36
	20 days	6	1.36
	30 days	6	1.33

## Data Availability

All data are provided in this manuscript.
